# MicroRNA-26b Regulates the Microglial Inflammatory Response in Hypoxia/Ischemia and Affects the Development of Vascular Cognitive Impairment

**DOI:** 10.3389/fncel.2018.00154

**Published:** 2018-06-08

**Authors:** Yuan-Cheng Kang, Li Zhang, Ying Su, Yue Li, Wen-Lei Ren, Wen-Shi Wei

**Affiliations:** Department of Neurology, Huadong Hospital, Fudan University, Shanghai, China

**Keywords:** microglia, miR-26b, vascular cognitive impairment, IL-6, inflammation

## Abstract

**Background:** Microglia play an important role in the central nervous system as immune cells and are often activated by post-ischemic injury. MicroRNAs are small endogenous RNAs affecting many complex cellular biological functions that are involved in neurodegenerative and cerebrovascular diseases. Previous studies have shown that microRNA-26b (miR-26b) is downregulated in BV-2 cells exposed to oxygen-glucose deprivation (OGD).

**Objective:** This study aimed to investigate how miR-26b regulates microglial activation and its neurotoxicity as well as the effect of miR-26b on vascular cognitive impairment (VCI).

**Methods:** Here, we used PCR to detect the mRNA expression of miR-26b and cytokines, western blot for the protein expression of cytokines, and the live/dead assay for neuronal apoptosis. In addition, we employed a luciferase assay to identify the possible target genes of miR-26b. Furthermore, we studied the effects of cerebral ischemia by bilateral common carotid artery occlusion (BCCAO) in rats. We used staining to identify neurons and microglia, and we tested cognitive function by the T-maze test.

**Results:** Our results showed that OGD activated microglia and increased the expression of interleukin (IL)-6 and other cytokines. Similarly, BCCAO activated microglia and increased the expression of IL-6 in the hippocampal CA1 area. We further found that miR-26b decreased the number of activated microglia and targeted IL-6. Moreover, miR-26b expression attenuated microglial activation, inflammation, neurotoxicity and VCI.

**Conclusion:** Our results suggested that miR-26b is involved in microglial activation and neurotoxicity in hypoxia/ischemia via IL-6. Therefore, increasing miR-26b expression may improve cognitive function.

## Introduction

Vascular cognitive impairment (VCI) is a cognitive disorder related to vascular brain injury, including mild cognitive impairment to dementia (Rincon and Wright, 2013). Meanwhile, vascular dementia (VD) is the second most common cause of dementia after Alzheimer’s disease (AD; Vos et al., 2016). Vascular mild cognitive impairment (V-MCI) is considered to be the early stage of VD and may be reversed (Gorelick et al., 2011). However, effective treatments for VCI are still not available.

Chronic microglial activation and neuroinflammation play important roles in the pathogenesis of dementia (Block et al., 2007). Microglia are the innate immune cells in the brain, accounting for approximately 10% of all brain cells. They have a high density in the hippocampus, olfactory telencephalon, basal ganglia, and substantia nigra (Block et al., 2007; Aguzzi et al., 2013). In the normal brain, resting microglia are activated by hypoxia, microbes, and other brain injuries (Town et al., 2005). The activated microglia play a major role in neuroinflammation by releasing neurotropic factor and devouring harmful substances to protect neurons (Block et al., 2007; Nayak et al., 2014). On the other hand, they produce a series of proinflammatory cytokines, including interleukin (IL)-6, interferon (IFN)-γ, tumor necrosis factor (TNF)-α and IL-1β, to diffuse the inflammatory response and to induce neuronal damage (Town et al., 2005). IL-6 is a major inducer of inflammation, which is involved in many cellular responses in the CNS such as gliogenesis, cell survival (Van Wagoner and Benveniste, 1999; Conroy et al., 2004). In patients, IL-6 is associated with many diseases. For example, patients with stroke showed significant elevations of CSF and serum IL-6 shortly after the ischemic event (Tarkowski et al., 1995; Smith et al., 2004). Circulating higher IL-6 contributes to incident dementia in patients with vascular risk factors (Miwa et al., 2016). Patients with VD also showed a high level of serum IL-6 (Dukic et al., 2016), which points to the inflammatory component in the development of VCI.

MicroRNAs (miRNAs) are related to many disorders in the central nervous system (CNS), and some miRNAs have been suggested as disease biomarkers and therapeutic targets (De Smaele et al., 2010). MiRNAs are evolutionarily conserved, endogenous small RNA molecules that are widely distributed in many animals, plants, and other organisms (Bartel, 2009). Mature miRNAs have no open reading frame and repress protein production by directly binding to the 3′-untranslated region (UTR) of the target gene to regulate gene expression (Ambros, 2004). Although the expression of miRNA varies in different tissues and organs, approximately 70% of miRNA is expressed in the brain (Filipowicz et al., 2008), which is closely associated with CNS development, neuronal differentiation, synaptic plasticity, and memory formation (Olde Loohuis et al., 2012; Neo et al., 2014; Schröder et al., 2014) (Van den Hove et al., 2014). It has been shown that miR-29, miR-15 and miR-107 are upregulated; while miR-124, miR-34 and miR-153 are downregulated in patients with AD (Delay et al., 2012; Lau et al., 2013). The decrease of miR-29b expression can increase apoptosis markers while disinhibiting BACE1 expression to contribute to the production of Aβ in AD (Hébert et al., 2008; Kole et al., 2011; Delay et al., 2012), suggesting that altered miRNA expression may affect the pathogenesis of AD through a variety of mechanisms. Although many researchers have studied the involvement of miRNAs in AD, information regarding miRNAs in VCI is rare. One study has shown that serum miR-93, miR-146a, miR-31 are significantly upregulated in VD compared to controls (Dong et al., 2015). MiRNAs also play important roles in the inflammatory response. For example, miR-146a can inhibit toll-like receptor signaling pathway-related proteins to suppress the inflammatory response (Li J. et al., 2014; O’Connell et al., 2012; Zhang et al., 2012). MiRNA-26b has mostly been studied in cancer, and it has been found that miR-26b is downregulated in breast cancer and that it can inhibit cellular proliferation (Li J. et al., 2014). Moreover, previous studies have shown that miRNA-26b is downregulated when exposed to oxygen-glucose deprivation (OGD; Zhang et al., 2012). However, it is unclear how miRNA-26b regulates the microglial inflammatory response in hypoxia/ischemia and how it affects the development of VCI.

Vascular lesions in VCI cause cerebral ischemia and hypoxia, which can induce the microglial activation that has been suggested to be associated with VCI (Jin et al., 2010). However, the regulation of miRNA-26b in microglial activation in VCI remains unclear. Therefore, this study aimed to investigate the effects of miRNA-26b on the inflammatory response in microglial activation and the possible mechanism of microglia in VCI.

## Materials and Methods

### Materials

All cell culture-related products were purchased from Gibco (Carlsbad, CA, USA). The following antibodies were used: goat anti-IL-6 and rabbit anti-IL-1β (Santa Cruz Biotechnology); rabbit anti-TNF-α (Abcam); rabbit anti-GAPDH (Cell Signaling Technology); all secondary antibodies were purchased from Santa Cruz Biotechnology. The miR-26b mimics (5′-UUCAAGUAAUUCAGGAUAGGU-3′ and 5′-CUAUCCUGAAUUACUUGAAUU-3′) and the negative controls (5′-UUCUUCGAACGUGUCACGUTT-3′ and 5′-*ACGUGACACGUUCGGAGAATT*-3′) as well as the miR-26b inhibitor (5′-ACCUAUCCUGAAUUACUUGAA-3′) and the negative control (5′-CAGUACUUUGUGUAGUACAA-3′) were synthesized by Genepharma (Shanghai, China).

### Cells and Animals

BV-2 cells (an immortalized cell line obtained from the primary cultured mouse microglia infected by the antiretroviral J2 carrying the oncogene v-raf/v-myc), SH-SY5Y cells (a subline cloned three times from the neuroblastoma cell line SK-N-SH (SK-N-SH → SH-SY → SH-SY5 → SH-SY5Y)), and 293T cells (a highly transfectable derivative of human embryonic kidney 293 cells and containing the SV40 T-antigen) were purchased from the American Type Culture Collection (Manassas, VA, USA). BV-2 cells and 293T cells were cultured in Dulbecco’s modified Eagle’s medium (DMEM), supplemented with 10% fetal bovine serum. SH-SY5Y cells were cultured in MEM/F12 with 10% fetal bovine serum. Newborn Sprague Dawley (SD) rats (within 24 h of birth) and Wistar adult male rats, aged 10–12 weeks, were provided by the Experimental Animal Center of the Chinese Academy of Sciences, Shanghai, China. All rat experiments were performed according to the guidelines of the National Institutes of Health for the Care and Use of Laboratory Animals. The protocol for this study was approved by the Committee on the Use of Live Animals in Teaching and Research, Fudan University, Shanghai.

### Microglial Cell Culture

Primary microglial cells were obtained according to previously described methods (Zhang et al., 2015). Briefly, brains of the newborn SD rats (<24 h) were rapidly removed under a microscope. After isolating the cortex from the brain in cold D-Hank’s solution, the cortex was digested by 0.125% trypsin, and the mixture was filtered. The precipitate was collected after centrifugation, and then the precipitate was resuspended in DMEM/F12 containing 10% fetal serum and cultured in 75-cm^2^ flasks coated with polylysine for 10 days, with the medium changed every 48 h. On day 10, after discarding the cell medium, the cells were incubated in a solution containing 0.5% lidocaine for several minutes, terminating the process with medium. After shaking the flasks at 180 rpm for 1 h, the primary microglial cells were obtained. The purity of the microglial cultures was evaluated by immunofluorescence assays using the Iba-1 antibody, and the positively stained cells were counted under a fluorescence microscope.

### Neuron Culture

Primary neurons were obtained from the hippocampus of newborn SD rats. After resuspending the precipitate in DMEM/F12, the cell mixture was cultured in a 6-well plate with 5–10 × 10^6^ cells per well. After 12 h, the medium was changed to Neurobasal medium containing 2% B27. After 48 h, the medium was replaced with Neurobasal medium containing cytarabine (10 μM); the medium was replaced every 2–3 days for 7 days. The purity of the neurons was assessed by an immunofluorescence assay using NeuN antibody, and the cells were counted under a fluorescence microscope.

### OGD

OGD of the microglia was performed according to previously described methods (Zhang et al., 2015). Briefly, microglia were cultured under anoxic (95% N_2_ and 5% CO_2_) conditions with serum/glucose-free DMEM at 37°C for 4 h. Then, the cells were transferred to a normal incubator (95% air and 5% CO_2_), and the medium was changed to serum-free medium containing glucose. After that, the cells were cultured for several hours, and then the supernatant was collected and mixed with new culture medium (1:1) to make microglial conditioned medium (MCM). Some of the MCM was mixed with IL-6 neutralizing antibody to neutralize IL-6. Additionally, after OGD was completed, some of the supernatant was collected immediately, and the activity of lactate dehydrogenase (LDH) in the supernatant was detected by an LDH kit (Beyotime, China). Meanwhile, after discarding the supernatant, radioimmunoprecipitation assay (RIPA) buffer was added to the cells, and the total protein was determined by the bicinchoninic acid assay. Then, the concentration of malondialdehyde (MDA, a product of lipid oxidation in living organisms) in the cells was determined by an MDA kit (Beyotime, China).

### Real-Time Polymerase Chain Reaction (PCR)

Total RNA was extracted using the total RNA Extraction Kit (Omega Bio-Tek, Norcross, GA, USA). The cDNA was synthesized using a FastQuant cDNA kit (Tiangen Biotech, Beijing, China). The real-time PCR system contained the following: 10 μL of SYBR Green premixture (2×), 0.5 μL of forward primer (10 μM), 0.5 μL of reverse primer (10 μM), 8.0 μL of water, and 1.0 μL of cDNA, for a total volume of 20 μL. The primers used were as follows: IL-6, forward: 5′-*ATGAAGTTTCTCTCCGCAAGA*-3′ and reverse: 5′-*CTAGGTTTGCCGAGTAGACCT*-3′; IL-1β, forward: 5′-*GACAAGCAACGACAAAATCCC*-3′ and reverse: 5′-*GAAGACAAACCGCTTTTCCATC*-3′; TNF-α, forward: 5′-*TACTGAACTTCGGGGTGATCG*-3′ and reverse: 5′-*TCCGCTTGGTGGTTTGCTAC*-3′; GAPDH, forward: 5′-*ATGGTGAAGGTCGGTGTGAA*-3′ and reverse: 5′-*TTACTCCTTGGAGGCCATGTA*-3′. For miRNA real-time PCR, total miRNA was extracted using a mirVana miRNA Isolation kit (Ambion). MiRNA cDNA was synthesized using an miRNA cDNA Synthesis Kit (CWBIO, Beijing, China), and miRNA real-time PCR was conducted using an miRNA qPCR Assay Kit (CWBIO, Beijing, China). The primers used were as follows: miR-26b, forward: 5′- *GCCGCTTCAAGTAATTCAGG*-3′ and reverse: 5′- *TATGGTTTTGACGACTGTGTGAT*-3′; U6, forward: 5′- *CAGCACATATACTAAAATTGGAACG*-3′ and reverse: 5′- *ACGAATTTGCGTGTCATCC*-3′. Cycle threshold (CT) values among the different samples were compared using the 2^−ΔΔ*Ct*^ method relative to GAPDH.

### Western Blot

Western blot was performed according to a previously established protocol (Zhang et al., 2015). Briefly, protein was extracted from cells or tissues by using RIPA lysis buffer. The lysate samples were separated by sodium dodecyl sulfate-polyacrylamide gel electrophoresis and transferred onto a polyvinylidene difluoride membrane. The membrane was blocked with 5% fat-free milk and then incubated with primary antibodies against IL-6 (1:500), IL-1β (1:1000), TNF-α (1:1000) and GAPDH (1:1000), respectively, at 4°C overnight. After washing three times, the membrane was incubated with horseradish peroxidase-conjugated secondary antibody for 1 h at room temperature. The proteins were visualized using the enhanced chemiluminescence reagents (Thermo). The protein bands were quantitated using ImageJ software, and the band intensities were normalized to those of GAPDH.

### Enzyme-Linked Immunosorbent Assay (ELISA) for IL-6

The concentration of IL-6 in the cell supernatant was assessed by ELISA, according to the manufacturer’s protocol (eBioscience). After the microglia were treated with OGD, the supernatant was collected, and the total protein level was normalized for each sample to conduct the ELISA for IL-6.

### Transfection

For 293T cells and BV-2 cells, the transfection reagent Lipofectamine 2000 (Invitrogen, Waltham, MA, USA) was used, according to the manufacturer’s protocol. Briefly, cells were seeded into 6-well plates and cultured for 24–48 h. The miRNA oligonucleotides were added to 250 μL of Opti-MEM, Lipofectamine 2000 was added to another 250 μL of Opti-MEM, and the separate solutions were incubated at room temperature for 5 min. Next, the two Opti-MEM solutions were mixed and incubated at room temperature for 20 min. The 500-μL mixture was added to each well, and serum-free medium was added. The cells were incubated for 6 h, and the medium was replaced with complete growth medium. For microglia, the transfection reagent Pepmute (SignaGen Laboratories, Rockville, MD, USA) was used, according to the manufacturer’s protocol. Briefly, before transfection, the cell culture medium was changed to new complete growth medium, and the cells were incubated for 1 h. The miR-26b or siIL6 oligonucleotides and reagent were added to Pepmute transfection buffer, and the mixture was incubated at room temperature for 15 min before it was added to the cells. The cells were incubated for 5 h, and then the buffer was replaced with complete growth medium.

### Live/Dead Staining of Neurons

Live/dead cell staining (Calcein-AM/propidium iodide (PI)) was conducted according to the manufacturer’s protocol (DOjinDO, Shanghai, China). Briefly, Calcein-AM (1 mM, dissolved in DMSO) and PI (1.5 mM, dissolved in doubly distilled H_2_O) were added to phosphate-buffered saline (PBS) to make the staining solution; the final concentrations were 2 μM and 4 μM, respectively. The neurons were washed with PBS, the staining solution was added to the cells, and the cells were incubated at 37°C for 15–30 min. After the staining reaction was stopped by washing with PBS, the cell membrane of the apoptotic cells was damaged so that PI could enter the cells, thus staining the nucleus a red color. In living cells, Calcein-AM could enter through the membrane, and the esterase could remove the AM radical to emit green fluorescence. The neurons were observed under a fluorescence microscope, and the numbers of live and dead cells were counted.

### Luciferase Assay

The 293T cells or BV-2 cells were seeded into 24-well plates at a cell density of approximately 5 × 10^4^ cells/well. The cells were cotransfected with the miR-26b mimics and the IL-6 mRNA 3′-UTR luciferase plasmid, or the miR-26b negative control and the IL-6 mRNA 3′-UTR luciferase plasmid by using the transfection reagent Lipofectamine 2000. After 6 h, the culture medium of the transfection complex was replaced with new culture medium, and the cells were incubated for an additional 24 h. The activities of firefly and *Renilla* luciferase were accessed by the Dual-Luciferase Reporter Assay System (Promega).

### Animal Bilateral Common Carotid Artery Occlusion (BCCAO) Surgery

Rats were restricted from food for 24 h and water for 4 h before they were anesthetized with 10% chloral hydrate (0.3 mL/100 g) by intraperitoneal injection. The rats were fixed on the operation platform with their neck exposed. After a midline incision was made, the skin and neck muscles were separated layer by layer, and then the left carotid artery was isolated and ligated with a surgical suture line. After 1 week, the right bilateral common carotid artery was ligated in the same way. Rats in the control group had their common carotid arteries isolated, but ligation was not performed.

### Stereotactic Injection

Rats were anesthetized with 10% chloral hydrate (0.3 mL/100 g) by intraperitoneal injection. MiR-26b lentivirus (Genepharma, Shanghai, China) (1 × 10^9^ TU/mL) was injected into the hippocampal CA1 area (anterior/posterior axis, −4 mm; medial/lateral axis, ±2 mm; dorsal/ventral axis, −3.3 mm) using a sterilized microsyringe at a rate of 0.2 μL/15 s, with a total volume of 2 μL/side, over 10 min. Rats in the control group were injected with control virus.

### Frozen Sections and Immunohistochemistry

Fluorescence immunohistochemical analysis was conducted based on previously established protocols (Zhang et al., 2015). After anesthetization, rats were perfused with 4% paraformaldehyde solution. The fixed brains were rapidly removed and dehydrated in sucrose solution. After embedding with optimal cutting temperature compound (Sakura, Japan), 40-μm-thick sections were cut by a microtome. The sections were incubated with the primary antibodies Iba-1 (Abcam) and IL-6 (Abcam), respectively, overnight and then incubated with Fluor green- or red-conjugated secondary antibodies for 2 h, avoiding light. Finally, the sections were observed under a fluorescence microscope.

### Nissl Staining

Brain sections were incubated in 1% toluidine blue solution for 40 min, followed by washing with water and immersion in 75%, 80% and 95% alcohol solution for 1 min, respectively, differentiation agent for 15 s, 100% alcohol solution for 1 min twice, and dimethylbenzene for 5 min twice, in sequence. Finally, the sections were sealed by Permount mounting medium and observed under a microscope.

### Electron Microscopy

For electron microscopy, the perfusion solution contained 4% paraformaldehyde and 2.5% glutaraldehyde. After perfusion, the hippocampal CA1 area was isolated at a volume of 1 mm^3^ under an upright microscope and soaked in fixation fluid. The samples were then fixed with 1% osmic acid and dehydrated with gradient acetone solutions (50%, 70%, 90% and 100%). After soaking in a mixture of acetone and Epon812, the samples were imbedded with Epon812 at 37°C overnight, 45°C for 24 h and 65°C for 24 h. Samples were cut into 70-nm slices, and the slices were stained with uranyl acetate, followed by lead citrate. Finally, the slices were observed under an electron microscope.

### T-maze Cognitive Assessment

T-maze spontaneous alteration was evaluated, as described previously (Zhang et al., 2015). The definition of spontaneous alteration is as follows: the rat entered one arm with all feet, returned to the starting position, and then entered the other arm with all feet. The number of alterations within 5 min was recorded. Before the experiment, the rats were allowed to explore the maze freely with all arms open for 10 min each day for 5 days. The rats were restricted from food until the body weight was maintained at 80%. In the adaptation phase, the rats were placed in one T-maze arm for 5 min with food in it, and then placed in the other arm. After adaption, the rats were trained in two sessions each day (9:00 am and 2:00 pm), including 11 conversions. During training, the rats were given food as a reward when they entered a different arm from last time. The rats were trained until they got at least nine correct choices of out 11 times on two consecutive trainings. The number of days for each rat to reach the standard was recorded. Five days later, the rats were put in the T-maze again to test the correct choices in 10 continuous conversions.

### Statistical Analysis

All data are presented as the means ± standard deviation. Statistical analysis was performed using SPSS15 statistical software. The main methods used were the *t*-test, χ^2^ test, and analysis of variance (ANOVA). For the biochemical results, the Student’s *t*-test and Pearson’s χ^2^ test were used; for multiple comparisons between groups, one-way ANOVA was applied. For qualitative variables, Pearson’s χ^2^ test was used. The ratio among the two groups was compared by the χ^2^ test. A *P*-value < 0.05 was considered significant.

## Results

### OGD Induced Microglial Activation and Reduced miR-26b Expression

**Figure 1:**
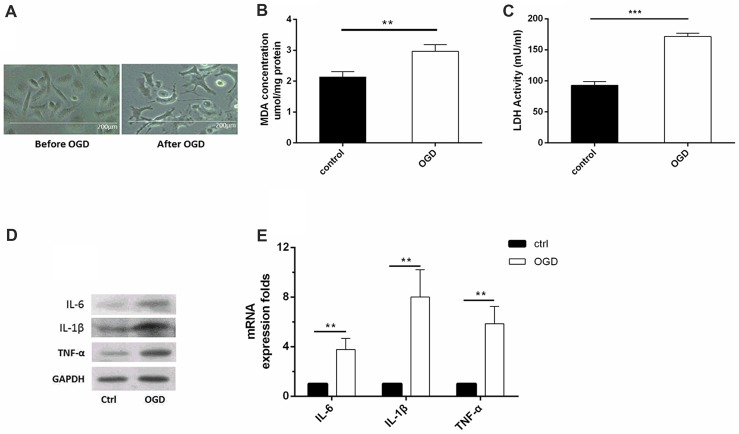
Oxygen-glucose deprivation (OGD)-induced microglial activation and release of inflammatory factors. **(A)** Images of microglia before and after OGD and reoxygenation for 48 h, as observed under a microscope. **(B)** Malondialdehyde (MDA) concentration after OGD. **(C)** Lactate dehydrogenase (LDH) activity after OGD. **(D–E)**. After OGD and reoxygenation for 48 h, the protein expression levels of interleukin (IL)-6, IL-1β and tumor necrosis factor (TNF)-α were detected by western blot, and the mRNA expression levels of these inflammatory factors were tested by real-time PCR. **P < 0.01; ***P < 0.001.

*In vitro*, we used OGD to induce hypoxia in microglia. Using microscopy, we observed that the structure of the cultured microglia after OGD showed enlarged cell bodies, shortened and polarized processes, and an ameboid shape in some microglia (Figure 1A). Meanwhile, the MDA level (Figure 1B) and the LDH activity (Figure 1C) were both significantly increased after OGD, compared to the control, indicating that the microglial cells were suffering from hypoxia. More importantly, the levels of inflammatory factors, including IL-6, IL-1β and TNF-α, were all significantly increased at 48 h after OGD, compared to the control (Figures 1D,E, Supplementary Figures S1A,B).

**Figure 2:**
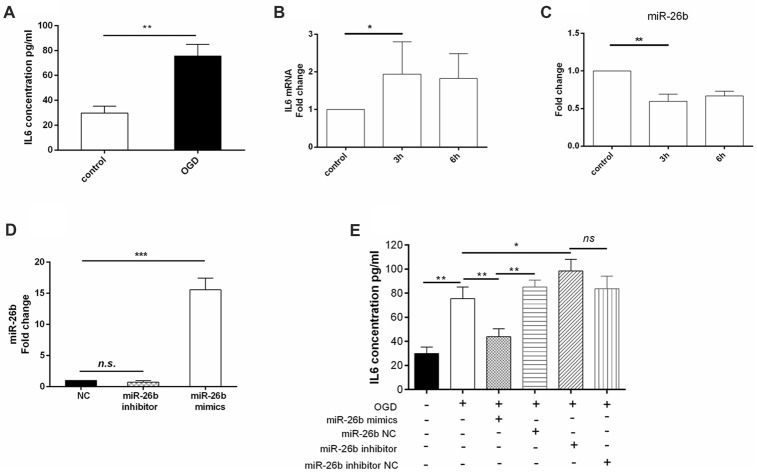
OGD induced changes of IL-6 expression in microglia, which was associated with downregulation of miR-26b. **(A)** After OGD and reoxygenation for 48 h, the concentration of IL-6 in the supernatant of microglia was evaluated by enzyme-linked immunosorbent assay (ELISA). **(B–C)** After OGD and reoxygenation for 3 h and 6 h, respectively, the mRNA levels of IL-6 and miR-26b were measured by real-time PCR. **(D)** Microglia were transfected with miR-26b mimics, and 48 h later, the level of miR-26b was detected by real-time PCR. **(E)** The concentrations of IL-6 in the supernatants of microglia that were transfected with miR-26b mimics, miR-26b NC, miR-26b inhibitor, and miR-26b inhibitor NC, respectively, were measured by ELISA after OGD treatment. *P < 0.05; **P < 0.01; ***P < 0.001; n.s., no significance.

Furthermore, we found that the IL-6 content (Figure 2A) was significantly increased by OGD as early as 3 h after OGD treatment (Figure 2B). In addition, the IL-6 mRNA level was increased (Figure 2B), consistent with a previous study in BV2 cells (Zhang et al., 2015). Meanwhile, we found that OGD significantly reduced the miR-26b expression after reoxygenation for 3 h and 6 h after OGD treatment (Figure 2C). In order to clarify the relationship between miR-26b and IL-6, we used miR-26b mimics that increased miR-26b expression (Figure 2D) and an miR-26b inhibitor that inhibited miR-26b function (there was no statistical difference in miR-26b expression; Figure 2D) to transfect the microglial cells and to examine their effect on IL-6 expression. After OGD, we found that IL-6 expression was significantly reduced in the miR-26b mimic groups, compared to that in the control group. In contrast, the miR-26b inhibitor significantly increased the IL-6 level (Figure 2E).

### IL-6 May be the Target of miR-26b

**Figure 3:**
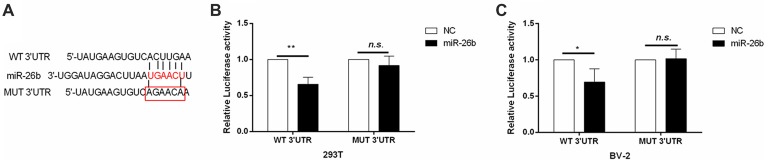
IL-6 is the target gene of miR-26b regulation. **(A)** Predicted miRNA binding sites in the 3′-untranslated region (UTR) of IL-6 mRNA. The wild-type or mutant IL-6 3′-UTR was cloned. **(B–C)** The wild-type (clone) and mutant type of the IL-6 mRNA 3′-UTR were constructed in the pZEX-MT01 vector, then transfected with miR-26b mimics and miR-26b NC, respectively, into 293T cells and BV-2 cells, and finally tested by a double luciferase reporter gene system. *P < 0.05; **P < 0.01; n.s., no significance.

Our previous study found that the IL-6 3′-UTR region might have an miR-26b binding site, suggesting that IL-6 may be a target gene of miR-26b (Zhang et al., 2015). We used the dual luciferase reporter assay system, embedded the clone (wild-type) or mutant IL-6 mRNA 3′-UTR (Figure 3A) into the pEZX-MT01 plasmid, and then transfected the plasmid and miR-26b mimics into the cells. In both 293T cells (Figure 3B) and BV-2 cells (Figure 3C), we found that the luciferase activity was inhibited in the wild-type by the miR-26b mimics, compared to that in the control group, while there was no significant difference in the luciferase activity of the mutant type between the miR-26b mimic groups and the control group.

### MiR-26b Could Inhibit Neuronal Apoptosis Induced by Microglial Activation in OGD

**Figure 4:**
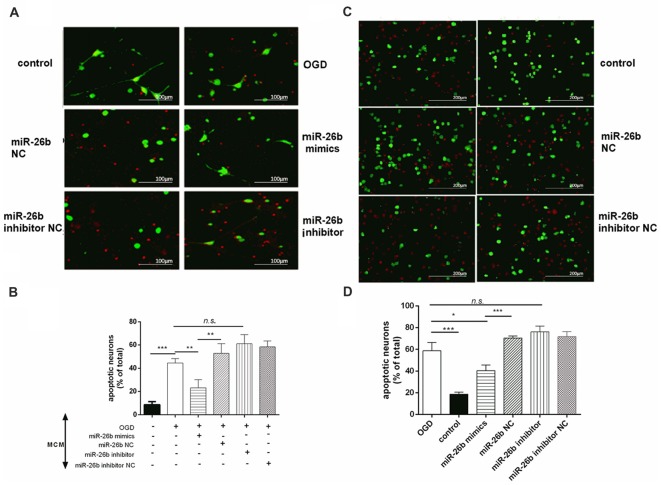
Microglial conditioned medium (MCM) induced neuronal apoptosis. **(A,C)** Primary neurons **(A)** and SH-SY5Y cells **(C)** were exposed to MCM for 48 h and stained by the live/dead assay: green fluorescence for surviving neurons and red fluorescence for apoptotic neurons. **(B,D)** The quantitative analysis of the proportion of apoptotic neurons in primary neurons **(B)** and in SH-SY5Y cells **(D)**. *P < 0.05; **P < 0.01; ***P < 0.001; n.s., no significance.

Microglia released large amounts of inflammatory cytokines after OGD. We collected the supernatant of microglial cells after OGD and exposed it to primary neurons for 48 h to test its effect on neuronal apoptosis. We showed that the OGD microglial cell supernatant induced neuronal apoptosis; however, the miR-26b mimics alleviated the neuronal apoptosis, while the miR-26b inhibitor aggravated the neuronal apoptosis in both primary neurons (Figures 4A,B) and SH-SY5Y cells (Figures 4C,D).

### The Effect of miR-26b on Microglia-Activated Toxicity, Which Was Partially Dependent on IL-6

**Figure 5:**
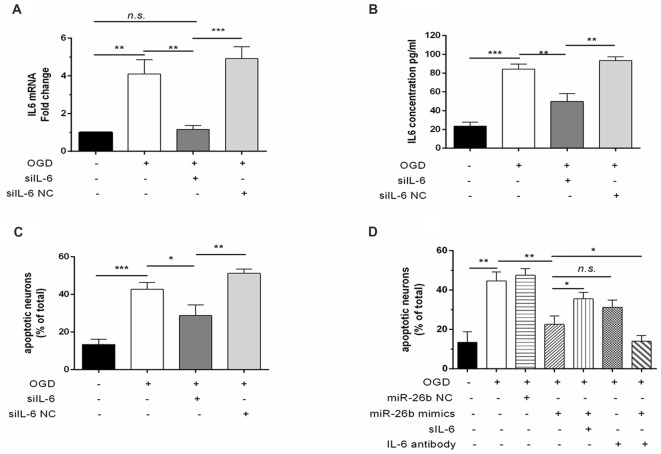
The effect of miR-26b on microglial neurotoxicity depended on the involvement of IL-6. **(A–B)** Microglia were transfected with IL-6 siRNA or negative controls; after OGD and reoxygenation for 48 h, IL-6 mRNA was detected by PCR **(A)** and the concentration of IL-6 was detected by ELISA **(B)**. **(C)** MCM was exposed to neurons, stained with the live/dead assay for the detection of neurons, and the proportion of apoptotic neurons was calculated. **(D)** Microglia were transfected with miR-26b mimics or NC; after OGD, recombinant IL-6 or IL-6 neutralizing antibody was added to the MCM; and after 48 h, neurons were stained with the live/dead assay, and the proportion of apoptotic neurons was calculated. *P < 0.05; **P < 0.01; ***P < 0.001; n.s., no significance.

To further clarify the role of IL-6 in regulating miR-26b in microglial neurotoxicity, we transfected IL-6 siRNA (siIL-6) into microglia and found that the IL-6 mRNA level (Figure 5A) and IL-6 content (Figure 5B) were significantly reduced after OGD, compared to the control, which imitated the effect of miR-26b, and the MCM, after transfection with siIL-6, could also lower the mortality of neurons after OGD (Figure 5C). Furthermore, we added recombinant IL-6 and IL-6 antibody to MCM and exposed the mixture to neurons. We found that the protective effect of miR-26b was decreased by recombinant IL-6 (Figure 5D), compared to the OGD+miR-26b group. However, after the IL-6 antibody neutralized IL-6, neuronal apoptosis was reduced, compared to the OGD group and there was no statistical difference with the OGD+miR-26b group.

### Microglial Activation, Neuronal Damage, and miR-26b Changes in the VCI Model

**Figure 6:**
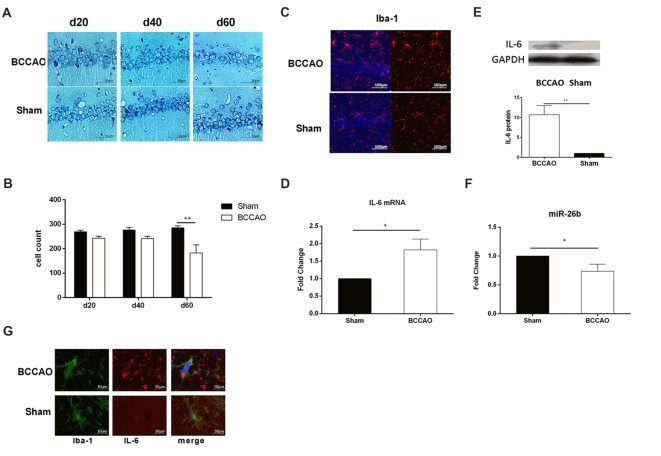
The vascular cognitive impairment (VCI) rat model showed microglial activation, neuronal damage, and miRNA changes in the hippocampal CA1 area. **(A)** Images of Nissl staining showing the hippocampal CA1 area at 20, 40 and 60 days after bilateral common carotid artery occlusion (BCCAO) treatment. **(B)** Quantitative analysis of the number of neurons in the hippocampal CA1 area at 20, 40 and 60 days after VCI treatment from three independent brain tissue slices. **(C)** Immunofluorescence images showing the hippocampal CA1 area after BCCAO, which were labeled with Iba-1 antibody. Red fluorescence indicated Iba-1-positive cells, while blue fluorescence indicated DAPI-labeled nuclei. **(D)** The mRNA level of IL-6 in the hippocampal CA1 area was measured by real-time PCR. **(E)** The protein level of IL-6 in the hippocampal CA1 area was measured by western blot. **(F)** The expression of miR-26b was detected by real-time PCR. **(G)** Immunofluorescence images showing the costaining of Iba-1 (green fluorescence), with IL-6 (red fluorescence), and DAPI (blue fluorescence). *P < 0.05; **P < 0.01.

We used BCCAO to induce the VCI rat model, according to a previous study (Zhang et al., 2015). From the Nissl staining images, we showed that there was a tendency toward a decrease of neuron number in the hippocampal CA1 area at days 20 and 40 and the neurons were distributed sparsely, which were significantly decreased at day 60 in the BCCAO group, compared to the sham group (Figures 6A,B). In contrast, the microglia were activated at day 60, with the cell body enlarged and the cell number increased in the BCCAO group, compared to the sham group (Figures 6C,D). Furthermore, we examined the IL-6 mRNA and protein levels in the hippocampus. The results showed that both the IL-6 mRNA (Figure 6E, Supplementary Figure S1C) and protein (Figure 6F) levels were significantly increased in the BCCAO group, compared to those in the sham group. Meanwhile, the miR-26b expression was significantly reduced in the BCCAO group, compared to the sham group (Figure 6G).

### MiR-26b Overexpression Could Alleviate Damage in the CA1 Area and Improve Learning and Memory Abilities in the VCI Model

**Figure 7:**
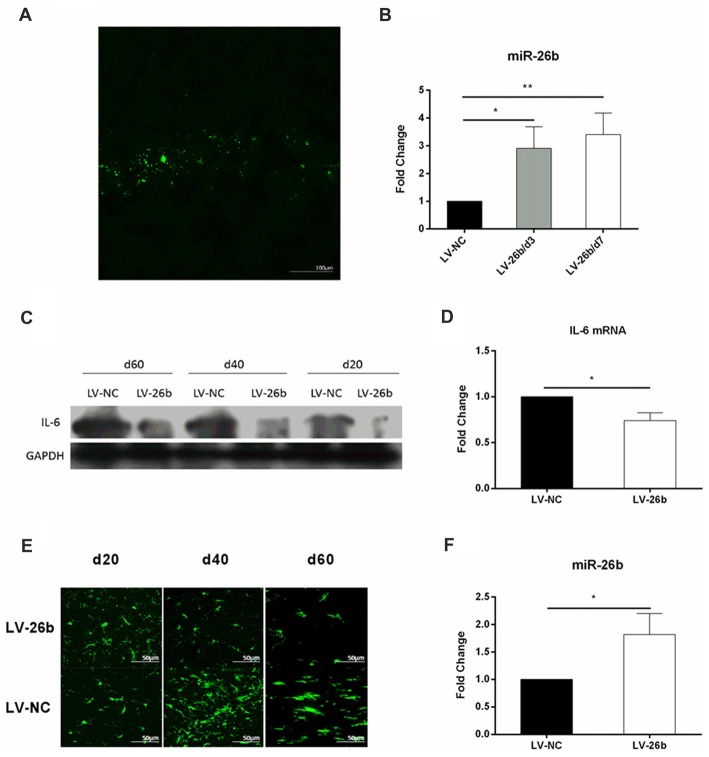
miR-26b overexpression in CA1 inhibited the expression of IL-6 and activation of microglia. **(A,B)** Lentiviral vector miR-26b (LV-26b) or negative control vector (LV-NC) carrying green fluorescent protein was stereotactically injected into the CA1 area at day 7, brain tissue was taken, brain slices were observed under a fluorescence microscope **(A)** and the expression of miR-26b was detected by real-time PCR at 3 and 7 days after viral injection **(B)**. **(C)** The hippocampal CA1 IL-6 protein level was measured by western blot on days 20, 40 and 60. **(D)** On day 60, the hippocampal CA1 IL-6 mRNA level was measured by real-time PCR. **(E)** Immunofluorescence images showing Iba-1 labeling (green fluorescence) at 20, 40 and 60 days after viral injection. **(F)** On day 60, the level of miR-26b was measured by real-time PCR. *P < 0.05; **P < 0.01.

To increase the level of miR-26b expression, we stereotactically injected miR-26b lentiviral vector (LV-26b) into the hippocampal CA1 area at day 2 after the VCI rat model was established. The lentiviral vector carries the green fluorescent protein gene, so green fluorescence was seen in brain slices at day 7 (Figure 7A). The miR-26 expression was significantly increased at 3 or 7 days after the LV-26b injection (Figure 7B). At days 60, the expression of miR-2b in LV-26b group was still higher than that in LV-NC group (Figure 7F). At days 20, 40 and 60 after the LV-26b injection, the IL-6 protein expression was lower in the LV-26b group than in the LV-NC group (Figures 7C,D, Supplementary Figure S1C). At 40 and 60 days after the LV-26b injection, the number of microglia was significantly decreased compared to that in the LV-NC group, while there was no significant change on day 20 (Figure 7E).

**Figure 8:**
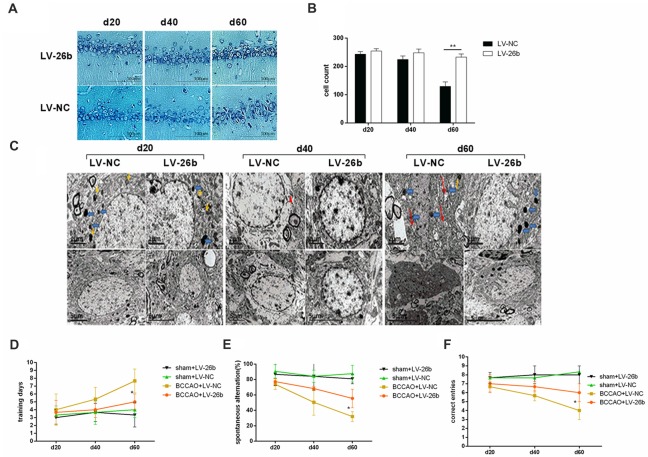
Increased miR-26b expression inhibited neuronal damage and improved cognitive impairment of the VCI model. **(A)** Images of Nissl staining showing the hippocampal CA1 area at 20, 40 and 60 days after BCCAO treatment. **(B)** Quantitative analysis of the number of neurons in the hippocampal CA1 area at 20, 40 and 60 days after viral injection treatment from three independent brain tissue slices. **(C)** The images from electron microscopy showing the ultrastructure of neurons in the CA1 area at 20, 40 and 60 days after viral injection. The yellow arrows indicate lipofuscin, the blue arrows indicate lysosomes, the red triangle indicates an autophagosome, and the red arrows indicate abnormal mitochondria. **(D)** The number of days to achieve the training standard. **(E)** The total number of spontaneous alternations. (**F**) The numbers of correct responses in the discrimination reaction. *P < 0.05; **P < 0.01.

Next, we studied the effect of LV-26b on the number of neurons and neuronal structure. Nissl staining showed that the number of neurons was significantly increased at day 60, compared to the LV-NC group; while there was an increasing trend at days 20 and 40 (Figures 8A,B). Furthermore, using electron microscopy, we found that the amounts of lipofuscin (yellow arrows) and lysosomes (blue arrows) were significantly increased at the early stage of VCI (day 20) in the LV-NC group, compared to the LV-26b group. At day 40, the neurons appeared significantly swollen and autophagosomes (red triangle) were observed in the LV-NC group, while neuronal swelling was alleviated in the LV-26b group. At day 60, neuronal apoptosis was observed, with cell body shrinkage, nuclear shrinkage, closed organelles, obviously swollen mitochondria (red arrows), and vacuolization with mitochondrial cristae reduced greatly in the LV-NC group; these manifestations were less severe in the LV-26b group (Figure 8C). Additionally, lipofuscin and lysosomes in the LV-NC group and lysosomes in the LV-26b group were observed at day 60.

Moreover, we used the T-maze to evaluate the learning and memory abilities of the rats. Our results showed that at day 60 after viral injection, the LV-26b group required fewer days to reach training standards as well as had more spontaneous alternation and a more correct discrimination reaction, compared with the LV-NC group, in the T-maze discrimination memory retention tests. However, there was no significant difference between the LV-26b and LV-NC groups in the T-maze learning and discrimination memory retention tests at 20 and 40 days after viral injection (Figures 8D–F).

## Discussion

In this study, we showed that microglia and its inflammatory reactions were activated by OGD and BCCAO. Furthermore, both OGD and BCCAO reduced miR-26 expression but increased IL-6 expression. However, increased miR-26b expression could attenuate microglial activation, inflammatory reactions, and cognitive function, which may occur via IL-6 regulation.

Microglia are the resident immune cells in the CNS that play an essential role in many CNS diseases, including AD, multiple sclerosis, and mental illness (Conroy et al., 2004; Prinz and Priller, 2014). After OGD treatment, the shape of the microglia was changed, including an enlarged and rounded cell body with deficient branches and some amoeboid cells. In primary microglia, OGD could also induce the release of large amounts of inflammatory cytokines, including IL-6. Previously, it has been shown that IL-6 and other inflammatory factors are increased after OGD treatment in BV-2 cells; in addition, the expression of many miRNAs is altered, including decreased miR-26b expression (Carlson et al., 1999; Zhang et al., 2012, 2015; Prinz and Priller, 2014). Increased miR-26b expression by its mimics in cultured microglia reduced the IL-6 level, while the miR-26b inhibitor increased IL-6 expression, suggesting that miR-26b activation is negatively correlated to IL-6 expression. Furthermore, we used a dual luciferase reporter system to demonstrate that miR-26b could bind to the IL-6 mRNA 3′-UTR rather than the mutant. These results indicate that miR-26b may inhibit transcription of IL-6 by binding to the IL-6 mRNA 3′-UTR, resulting in reduced IL-6 production.

The activated microglia induced by OGD could increase neuronal apoptosis, which was reversed by the increase of miR-26b expression. Reducing IL-6 expression by neutralization with its antibody or IL-6 siRNA could decrease neuronal apoptosis. These results suggested that miR-26b affected neuronal apoptosis under OGD treatment via IL-6. IL-6 is considered to be a proinflammatory cytokine in the CNS, but its function is complicated and controversial. IL-6 has a neuroprotective effect in N-methyl-D-aspartate-induced neuronal apoptosis by promoting nerve growth factors (Carlson et al., 1999). Conversely, IL-6 transgenic mice have shown decreased neuronal function and increased neuronal apoptosis (Conroy et al., 2004; Li S. J. et al., 2014) also have found that IL-6 can promote neuronal apoptosis in brain hypoxic/ischemic injury. In our study, we reported that IL-6 could increase neuronal apoptosis. In the VCI animal model, we found that the microglia were activated, IL-6 production was increased, and the expression of miR-26b was reduced in the hippocampal CA1 area; these data were consistent with the OGD experimental results. A previous study has reported that neuronal apoptosis in the hippocampal CA1 area is gradually increased during the process of ischemia (Ohtaki et al., 2006; Farkas et al., 2007). Consistently, we revealed that there was a mild decrease in the number of neurons at days 20 and 40, and there were few neurons at day 60. In addition, at the early stage of ischemia, the amounts of lysosomes, lipofuscin and cytolysosomes were increased and neuronal apoptosis was significantly increased, suggesting that neuronal damage was gradually exacerbated. However, increased miR-26b expression in the hippocampal CA1 area could reduce brain inflammation as well as neuronal damage. Yet, because miRNA can have many targets and the downstream target gene of miR-26b could also be MAPK and so on, miR-26b overexpression may also change the expression of these targets, thus affecting cellular function. Therefore, further research is needed.

## Conclusion

In this study, we found that OGD could induce activation of microglia. In addition, activated microglia could downregulate miR-26b expression. The reduction of miR-26b expression was related to the activation of microglia, the generation of IL-6, and the neurotoxic effects of activated microglia. Moreover, increased miR-26b expression in the hippocampal CA1 area in the VCI rat model could inhibit the activation of microglia and the production of IL-6 as well as reduce neuronal apoptosis, thus alleviating the cognitive impairment. Therefore, our study may provide a new target for studying the mechanism of VCI as well as the possible target for treating patients with VCI.

## Author Contributions

Y-CK, LZ, YS, and W-SW: conceived and designed the experiments. Y-CK, YS, YL and W-LR: performed the experiments. Y-CK and LZ: analyzed the data. Y-CK, LZ, YS, YL, W-LR and W-SW: contributed reagents/materials/analysis tools. YCK, LZ and W-SW: wrote the article.

## Conflict of Interest Statement

The authors declare that the research was conducted in the absence of any commercial or financial relationships that could be construed as a potential conflict of interest. The reviewer CY declared a shared affiliation, though no other collaboration, with the authors to the handling Editor.

## Supplementary Material

FIGURE S1 After oxygen-glucose deprivation (OGD) and reoxygenation for 48 h, the protein expression levels of interleukin (IL)-6 **(B)**, IL-1 β **(A)** and tumor necrosis factor (TNF)-α **(B)** were detected by western blot. In rats, the protein level of IL-6 in the hippocampal CA1 area was measured by western blot **(C)**.
