# Supplementary figures and images for: MicroRNA-26b Regulates the Microglial Inflammatory Response in Hypoxia/Ischemia and Affects the Development of Vascular Cognitive Impairment
Source: Front Cell Neurosci. 2018 Jun 8;12:154. doi: 10.3389/fncel.2018.00154 (PMC6002499; doi:10.3389/fncel.2018.00154)

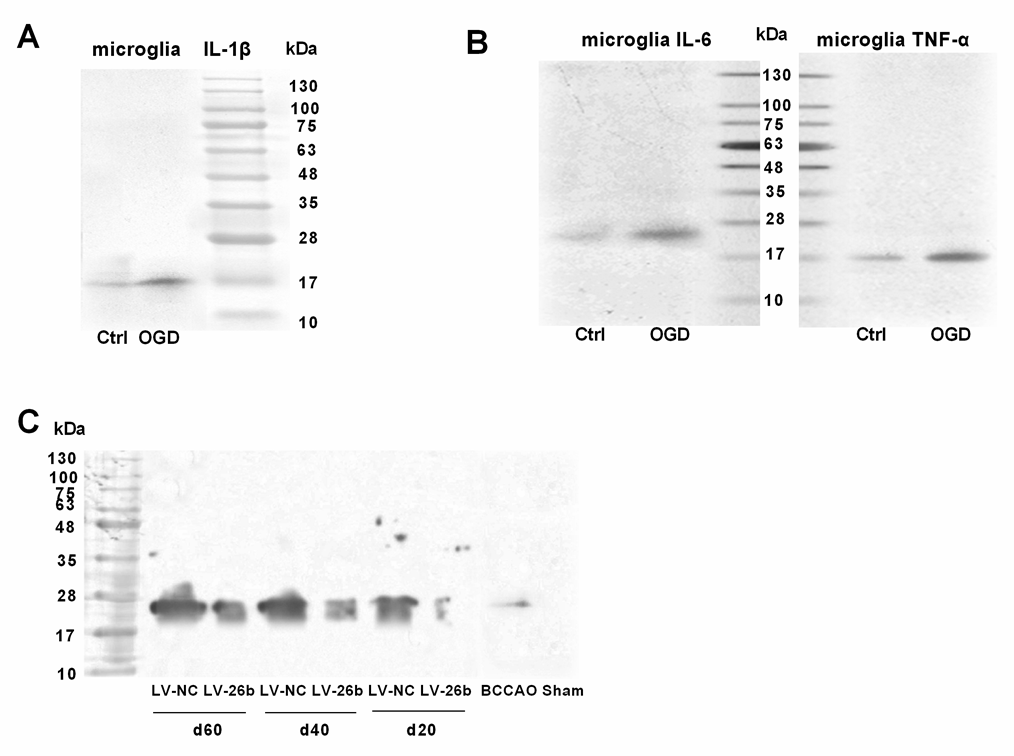

Supplement: FIGURE S1 — After oxygen-glucose deprivation (OGD) and reoxygenation for 48 h, the protein expression levels of interleukin (IL)-6 (B), IL-1 β (A) and tumor necrosis factor (TNF)-α (B) were detected by western blot. In rats, the protein level of IL-6 in the hippocampal CA1 area was measured by western blot (C). [file Image_1.TIF]
